# One Pressure, Many Targets: Left Ventricular Filling Pressure as a Candidate Effect Modifier for Non-Invasive Ventilation in Acute Coronary Syndromes

**DOI:** 10.3390/life16101626

**Published:** 2026-09-28

**Authors:** Marek Tomala

**Affiliations:** 1 Faculty of Medicine and Health Sciences, University of Applied Sciences in Nowy Sącz, 33-300 Nowy Sącz, Poland; m.tomala@intercard.net.pl; 2 Center for Invasive Cardiology, Electrotherapy, and Angiology, Clinical Research Center Intercard, Al. Beliny-Prażmowskiego 60/4, 31-514 Krakow, Poland

**Keywords:** non-invasive ventilation, continuous positive airway pressure, cardiogenic pulmonary edema, acute coronary syndrome, heart-lung interaction, transmural pressure, ventricular interdependence, ischemic mitral regurgitation, alveolar recruitment

## Abstract

Non-invasive ventilation relieves acute cardiogenic pulmonary edema and reduces intubation. Whether it saves lives is disputed; restricting the pooled record to the least biased trials leaves neither endpoint significant. My hypothesis: raising pleural pressure subtracts venous return; whether that subtraction is banked as unloading or paid as lost output depends on preload responsiveness, for which left ventricular filling pressure is a candidate effect modifier and not a measure. One radiographic label may contain two gas-exchange phenotypes, proposed for groups, not patients: carbon dioxide accumulates far more slowly than an ischemia-driven presentation floods the alveolus, and the remodeled ventricle arrives hypercapnic. Applied pressure inflates flooded air spaces without removing lung water, lowers left ventricular transmural pressure, and reaches two cardiac submechanisms. The left ventricular arm follows the sign rule; the renal arm follows it only through volume status; and in right ventricular infarction, the recorded filling pressure misleads, while the pulmonary arm does not reverse. In acute coronary syndromes, the first prediction fails if transmural pressure falls no more in the elevated than in the normal stratum, or if stroke volume index does not fall in the latter. The outer boundary is the requirement for emergency reperfusion, a limit inferred rather than measured directly.

## 1. Introduction

A patient drowns in an ultrafiltrate of his own plasma. Acute cardiogenic pulmonary edema develops within minutes, before troponin rises or a catheter is placed, and the first reflex is to seal a mask over the nose and mouth and use positive pressure to open congested alveoli. Relief arrives before the diagnosis. The work of breathing falls, oxygen saturation rises, and the patient settles.

Then the puzzle begins. The two endpoints do not rest on equal evidence. Pooled across randomized trials, non-invasive ventilation probably reduces intubation, RR 0.49 (95% CI 0.38 to 0.62, moderate quality of evidence), and may reduce hospital mortality, RR 0.65 (95% CI 0.51 to 0.82, low quality of evidence) [1].

Restricting the analysis to trials at low risk of bias leaves neither effect significant, although both intervals still include benefit: mortality across 7 trials with 1505 participants, RR 0.81 (95% CI 0.61 to 1.06), and intubation across 6 trials with 1491 participants, RR 0.85 (95% CI 0.55 to 1.32) [1].

Gray et al. randomized 1069 patients to standard oxygen or non-invasive ventilation and found no difference in 7-day mortality (9.8% versus 9.5%, *p* = 0.87) [2]. Intubation in the standard oxygen arm was rare (2.8%) [2]. Respiratory status improved. Survival did not. The error may lie in the averaging.

One radiographic pattern of alveolar congestion arises from two different ventricles: one that becomes ischemic and stiffens within minutes, and one that remodels and decompensates over days. The average hides that difference. Section 2 and Section 3 develop it. One term first. The target condition here is acute cardiogenic pulmonary edema precipitated by an acute coronary syndrome, with or without infarction. I write acute coronary syndrome for the precipitant throughout, and edema complicating infarction only where a cited cohort was restricted that way.

Hypothesis. Raising pleural pressure lowers venous return. What that loss costs depends on preload responsiveness. Throughout this paper, left ventricular end-diastolic pressure (LVEDP) enters as a candidate effect modifier of the response to applied pressure, not as a measure of preload responsiveness, the functional state in which stroke volume falls when venous return falls. Filling pressure reports loading conditions and chamber compliance, and no threshold of it establishes that state. The two part company wherever a high recorded pressure accompanies an underfilled, preload-responsive left ventricle: right ventricular infarction, biventricular failure, tamponade-like physiology, and strong ventricular interaction, the mechanism Section 4.7 develops. In those states the construct predicts payment rather than banking, so the recorded pressure misleads in a direction it names in advance. The parameter is left ventricular end-diastolic pressure. Where a source did not measure it, I name the surrogate that source used: pulmonary artery occlusion pressure in the invasive cohorts, the mitral inflow to annular velocity ratio with left atrial volume at the bedside. Neither is interchangeable with end-diastolic pressure, and Section 7 gives how far the bedside estimate falls short. Filling pressure is not the only candidate axis: Hieda uses end-systolic elastance rather than filling pressure, and places preserved ejection fraction on the preload-responsive side and severe infarction on the afterload-dependent side [3]. The stiff, acutely ischemic ventricle of Section 2 sits on that border, which is why the model is stated between strata and tested as a prespecified stratum by treatment interaction. Preload responsiveness has been shown in chronic heart failure and never in a cohort restricted to acute coronary syndromes. The model therefore predicts a difference in treatment effect between strata; it claims no threshold in filling pressure and says nothing about an individual patient within a stratum. In the stiff ventricle that fills at high pressure, the lost return is banked: the chamber performs less work at the same output. In the normally filling ventricle, the arterial response to a falling stroke volume cancels the afterload gain, and the same loss appears as lost flow. The same arithmetic applies at the renal vein. In right ventricular infarction, the recorded left ventricular filling pressure can be high while the left ventricle is underfilled and preload responsive. That dissociation between the candidate modifier and the state it stands in for is the model’s most direct test.

The model generates four predictions, none of them about an individual patient. Table 1 pairs each with the measurement it requires and the observation that would refute it. Figure 1 maps the whole argument.

Band one states the puzzle. In trials at low risk of bias the effects on intubation and mortality lose significance (Section 1). The inset gives this paper’s reading of that loss, inferred and not measured: an effect that changes sign between strata can cancel in an unstratified average.

Band two puts two gas-exchange phenotypes under one radiographic picture. The speed of alveolar flooding sets the gas; etiology predicts it only through that speed, and the group distributions overlap across most of their range (Section 2 and Section 3).

Band three splits one applied pressure four ways (Section 4). The lung branch does not reverse; it was measured in dogs and in acute left heart failure, and in edema complicating infarction only its effect on oxygenation was recorded. The left ventricular branch changes sign between filling-pressure strata in three cohorts from another population. The renal branch runs two ways: the first seen in a mixed intensive care cohort under invasive ventilation, the second inferred, with volume status deciding which prevails. The mitral leak of circumflex origin is inferred, and interdependence in right ventricular involvement has an unproven net effect.

The panels on the right hold the sharpest test, right ventricular infarction. A high recorded filling pressure can sit beside an underfilled, preload-responsive left ventricle, so the candidate modifier and the mechanism point in opposite directions (Section 4.7). Emergency reperfusion marks the outer boundary, read off trial exclusion criteria rather than measured (Section 1 and Section 5). The bottom band refers to Table 1 for the observations that would refute the construct.

Only the phenotype panel and the lung branch’s effect on oxygenation rest on cohorts or subgroups restricted to acute coronary syndrome or edema complicating infarction. Created in BioRender. Tomala, M. (2026).

One objection deserves an answer before the others. The trials that built this evidence base were designed around acute cardiogenic pulmonary edema, and acute coronary syndromes were not their target. They excluded patients who needed an immediate lifesaving procedure, including primary percutaneous coronary intervention, before randomization [2].

The clinical practice guidelines draw the same line from the other side. They recommend either bilevel NIV or CPAP for acute respiratory failure due to cardiogenic pulmonary edema, a strong recommendation on moderate certainty of evidence, and state that this recommendation does not apply to acute coronary syndrome or cardiogenic shock [4].

## 2. The Coronary Substrate: Why an Ischemic Ventricle Floods in Minutes

To begin with, an ischemic ventricle does not fail slowly; first it stiffens and then it weakens, and both faults push the same pressure upward. Relaxation is an energy-consuming process, driven by the reuptake of calcium into the sarcoplasmic reticulum, and the fall in oxidative energy supply at the onset of ischemia hampers it early [5]. In the ischemic cascade, dysfunction during diastole precedes dysfunction during systole; electrocardiographic change and chest pain occur later in the sequence [6]. When a segment fails it no longer contributes to ejection, and residual volume is added to a chamber that has already lost the capacity to accept blood at low pressure, so end-diastolic pressure increases and, the mitral valve being open throughout diastole, is transmitted to the pulmonary veins without obstruction. Thus a problem of contraction becomes a problem of gas exchange within a few beats.

In experimental preparations, lymph flow rises slowly and reaches a new steady state within about four hours of a sustained rise in capillary pressure [7]. Filtration rises at once, so when capillary hydrostatic pressure jumps within a few beats, filtration exceeds clearance immediately [7,8], and the air spaces fill immediately after the interstitial reservoir is saturated, with the barrier still intact.

Only at far higher transmural loads does the barrier itself fail, a threshold measured in anesthetized rabbits [9]. A patient who has carried a similar pressure for months stays dry, because the interstitial matrix expands and holds the excess as a gel, and flooding waits until left atrial pressure passes about 25 mmHg and overwhelms it [7]. The pressure height determines whether transmural unloading will later be banked. The speed at which it arrives sets whether the alveolus floods.

The time factor may therefore show in the arterial sample, although that sample also reports ventilation, dead space and the oxygen given (Section 3). Carrillo-Alemán and colleagues measured the split in 1009 patients ventilated non-invasively for acute heart failure, 403 of them with an acute coronary syndrome and infarction: that group began ventilation at a mean arterial carbon dioxide tension of 44 ± 18 mmHg against 51 ± 22 mmHg in the rest, *p* < 0.001 [10]. Lactate was higher and bicarbonate lower, differences the authors judged statistically significant but not clinically relevant, and ejection fraction was lower [10]. My position emphasizes directionality, not magnitude: edema precipitated by an acute coronary syndrome arrives less hypercapnic and more lactatemic, and that was observed in patients rather than assigned to trials. That cohort also reports no mortality difference by trigger: 16.6% with an acute coronary syndrome and infarction versus 14.9% without, *p* = 0.478 [10]. Every patient in it received ventilation, so it compares prognosis between two etiologies under the same treatment and cannot test whether the size of the benefit depends on the trigger.

The relationship is not universal. Where an ischemic ventricle is given days rather than minutes, as in acute coronary syndromes complicated by acute systolic heart failure, mean carbon dioxide tension can sit well into the hypercapnic range [11]. In this reading, speed shapes the gas and etiology predicts it only through the speed it implies. Phenotype is a surrogate for that speed, a gradient rather than a threshold, so Section 3 does not read the cutoff near 45 mmHg as a population description.

## 3. What the Blood Gas Reports

A flooded alveolus destroys oxygen transfer long before it disturbs carbon dioxide clearance. Carbon dioxide diffuses more readily than oxygen, but a flooded alveolus is not a thickened barrier. It is a shunt: in acute cardiogenic pulmonary edema, excess interstitial and alveolar fluid produces the shunt effect that reduces gas exchange [8]. No gas crosses it.

Petersson and Glenny supply the asymmetry a clinician reads on the sample. Hypoxemia caused by shunt responds poorly to supplemental oxygen, and with increasing shunt fraction the effect of raising the inspired fraction falls away, so that for very large shunts even an inspired fraction of 1.0 has little effect on arterial oxygen tension [12]. Oxygen cannot improve alveolar oxygen tension in unventilated units, and the small extra oxygen added to blood passing through open units does not offset the shunted blood [12]. Carbon dioxide behaves the opposite way, because the drive to hold arterial carbon dioxide tension normal increases ventilation of the units that remain open, at the cost of higher work of breathing [12]. The same holds for wasted ventilation in high ventilation-perfusion regions, where the usual result is a higher minute ventilation rather than hypercapnia [12].

Read this way, the two phenotypes become a statement about the pump and about time. In the flash, ischemic pattern, flooding outpaces any rise in carbon dioxide and the sample returns to eucapnia or arrives less hypercapnic over a lactic acidosis [10]. In the second, ventilatory reserve plausibly erodes before the alveoli drown, and the sample returns hypercapnia with respiratory acidosis [10]. The sample is not a pure clock. Arterial carbon dioxide also reports alveolar ventilation and dead space: hypoventilation raises it in any lung, a large dead space raises it in chronic obstructive pulmonary disease even at twice the normal minute ventilation, and supplemental oxygen can hold arterial oxygen tension above 90 mmHg while hypoventilation drives carbon dioxide up [12]. The clinical record carries a matching signal. Stratified by admission gas status, pooled hospital mortality separates an apparent benefit in the eucapnic stratum, RR 0.41 (95% CI 0.27 to 0.63), from an estimate near the null in the hypercapnic one, RR 0.82 (95% CI 0.65 to 1.03), *p* = 0.005 for the subgroup difference [1]. I report it once and decline to lean on it: whole trials were assigned to strata by their mean baseline carbon dioxide, not patients by their own gas [1]. An ecological contrast, nothing more.

A trial-level association between the proportion of ischemic edema and the size of the mortality effect points the same way, and it is not my priority. Weng and colleagues put that hypothesis first, reporting, for continuous positive airway pressure against standard therapy, RR 0.92 (95% CI 0.76 to 1.10) at a 10% ischemic fraction against 0.43 (95% CI 0.17 to 1.07) at 50% [13]; my own meta-regression reproduces the direction on a narrower definition of the covariate, and I cite it for direction alone [14]. That work highlighted the distinction between acute and chronic ischemia: the pooled covariate mixed acute coronary syndromes with chronic ischemic heart disease and prior infarction acting as precipitants, so I included only ischemic events confirmed within 0 to 72 h of admission or randomization [14]. The Fifth Universal Definition of Myocardial Infarction underlines the same distinction in its own tables, placing acute heart failure among the mechanisms of acute myocardial injury and chronic coronary syndrome among the mechanisms of chronic myocardial injury [15]. That covariate needs one disclosure: for the largest trial I took the coronary proportion from the endpoint table rather than from the baseline characteristics, so a variable measured after randomization entered the model as a study-level covariate, and the direction of the resulting bias cannot be stated without refitting the model [14]. Weng and colleagues also record that the largest trial carried less than half the weight in the analyses favoring ventilation [13].

The occurrence of severe hypercapnia above 60 mmHg is associated with a longer duration of ventilation, a more severe clinical course, and a prolonged intensive care unit stay, whereas hypercapnia above 45 mmHg alone does not increase the frequency of intubation [16]. It may therefore be regarded more as an indicator of organizational burden than of the eventual outcome.

## 4. One Applied Pressure, Many Targets

### 4.1. The Stimulus and Its Branches

Non-invasive ventilation is not a pharmacy of separate agents. It is one mechanical stimulus whose consequences branch. Positive-pressure ventilation raises pleural pressure, which lowers venous return and preload [17] and lowers left ventricular transmural pressure [18], while right ventricular afterload follows lung volume instead, along a U-shaped curve on which pulmonary vascular resistance rises at both collapse and overdistension [17]. That rule sorts the branches into two families. One follows pleural pressure and obeys the sign rule of Section 4.3: left ventricular transmural load, the subendocardial gradient derived from it, right ventricular preload, renal venous pressure. The other follows lung volume: alveolar recruitment, the oxygenation it buys, and right ventricular afterload. The first two keep their direction at any lung volume. Right ventricular afterload does not, because the U-shaped curve gives it a minimum [17], so its sign turns with lung volume and not with filling pressure. The modes are not interchangeable, and the paper is written for continuous positive airway pressure by mask. The branch that changes sign follows the end-expiratory pressure, which bilevel support delivers as well, adding an inspiratory pressure on top of it. At the same end-expiratory pressure of 5 cmH_2_O, bilevel support narrowed the esophageal swing more than continuous pressure alone in six patients with acute cardiogenic pulmonary edema and no acute infarction, though significance was reported only against spontaneous breathing, with no direct test between the two modes reported (Section 4.8) [19]. Within the model, the two modes are therefore expected to share the direction of the sign rule at the same end-expiratory pressure but not its size, since the added inspiratory pressure should lower transmural pressure further; that equivalence of direction is inferred, not measured. Invasive ventilation is a third thing, since the pressure arrives through a tube and the machine takes over the work of breathing, so the earliest of the three stratified cohorts of Section 4.3, ventilated that way, carries a transfer and not the same treatment.

Three levels of evidence run through what follows and through Table 1. Measured in this population means recorded in a cohort or subgroup restricted to humans with an acute coronary syndrome or with edema complicating infarction; measured in another population means recorded elsewhere, in humans or in animals, with the source population named in the sentence that carries the finding; inferred means neither, and it takes an explicit hedge. The branches follow one at a time, beginning with the lung. I call the result speculative, and the word is meant strictly. Nobody has measured transmural pressure, filling pressure, stroke volume and the response to a mask in one group of patients with an acute coronary syndrome and pulmonary edema. What follows is assembled from patients ventilated through a tube, from stable chronic heart failure and dilated cardiomyopathy, from healthy volunteers, from dogs and pigs, from de novo hypoxemic respiratory failure, and from anesthesia and weaning, which differ in effort, in pressure transmission, in ventricular function, in volume state and in the pressure applied. The tiers say which of those each branch rests on, and Section 8 weighs what the transfer costs.

### 4.2. The Lung: What Pressure Does to a Flooded Air Space

Positive pressure does not dry the lung, and the experiment most often cited for the opposite claim shows the opposite.

Malo, Ali and Wood ventilated the right and left lungs of dogs separately, produced edema with intravenous oleic acid until shunt in each lower lobe rose from 0.10 to 0.47, then raised end-expiratory pressure in one lung alone from 3 to 13 cmH_2_O [20]. Ten minutes later mean lobar shunt in the treated lobe had fallen to 0.06 against 0.48 in the untreated lobe, yet extravascular lung liquid did not differ between the lobes and the fraction of pulmonary blood flow reaching the treated lobe did not change, so pressure removed no water and diverted no perfusion [20]. What remained is the mechanism worth claiming: alveolar liquid moved into the compliant perivascular interstitium, where cuff liquid measured 3.8 ± 2.8 mL/g with end-expiratory pressure against 2.4 ± 1.7 mL/g without it, and light microscopy found 77.8 ± 9.0% of alveoli flooded without it against 22.2 ± 11.8% with it [20].

One detail sharpens the word recruitment. Pressure did not distend individual alveoli: mean linear intercepts of flooded alveoli did not differ between lobes [20]. Units were recruited, not stretched. Two boundaries: the model is permeability edema from oleic acid, so the transfer is an inference, and cardiac output fell once pressure was applied [20].

Relocation and recruitment leave one thing untouched. The alveolar-capillary barrier itself is the part pressure cannot repair [9]. Pressure only lowers the demand on it, by raising alveolar pressure against capillary transmural pressure [9] and by reducing the large negative swings that would otherwise widen pulmonary vascular transmural pressure and worsen leak [21]. The mechanical consequence is measurable. In nine patients in acute left heart failure with a pulmonary artery occlusion pressure of at least 18 mmHg, dynamic compliance rose from 60 ± 10 to 87 ± 20 mL/cmH_2_O and work of breathing fell from 18 ± 3 to 12 ± 2 J/min at 10 cmH_2_O of continuous positive airway pressure [22]. Cardiac index and stroke volume index were unchanged [22]. The loop in which hypoxemia raises drive and drive raises the pressure cost of every breath is broken, and nothing is paid for it in forward output.

Support appears to help regardless of the trigger. In a cohort ventilated for cardiogenic pulmonary edema, weaning succeeded in 90.6% of infarction-precipitated episodes and 90.8% of the rest (*p* = 0.950) [23]. Recruitment itself was not measured, and oxygenation rose in both groups, so the comparison reflects the course under support, not the mechanism. One finding runs the other way: intubation after weaning was more frequent in the infarction group (7.5% vs. 0.7%, *p* = 0.016 [23]). Weaning did not differ; what followed it did. Six-hour treatment failure was 24% on continuous positive airway pressure versus 50% on control, in one of the 24 trials pooled in the anchor synthesis rather than in an independent study [1,24]. If the lung response does not separate the phenotypes, the ventricle is the candidate place where they part.

### 4.3. The Left Ventricle and the Pressure That Actually Loads It

Afterload is systolic wall stress, and the operative pressure term is transmural, not intracavitary and not airway [18]. Pressure outside the heart and pressure inside its cavity subtract, and the muscle works against the difference, so raising pressure in the chest unloads the chamber without changing the pressure it generates.

The cleanest human demonstration comes from chronic heart failure, not acute ischemia; the distinction matters. Naughton and colleagues studied awake, supine, normoxic, eucapnic patients with chronic congestive heart failure and a mean ejection fraction of 17.9 ± 2.4%. At 10 cmH_2_O of continuous positive airway pressure, inspiratory esophageal pressure amplitude fell from 11.1 ± 1.1 to 7.5 ± 1.1 mmHg and systolic left ventricular transmural pressure from 116.0 ± 5.3 to 110.3 ± 4.5 mmHg, a fall of 5.7 mmHg, *p* < 0.025 [25]. Heart rate fell by 5.2 beats per minute, and the product of transmural pressure and heart rate, a surrogate for myocardial oxygen consumption, dropped by 11% (*p* < 0.005), while cardiac index and stroke volume index did not change [25]. The ventricle did less work at the same output.

The experiment that bounds the claim sits inside the same paper. In the healthy comparison group, systolic esophageal pressure rose by 4.1 mmHg (*p* < 0.005), compared with 5.3 mmHg in heart failure (*p* < 0.001), so the pressure was transmitted in both [25]. Yet systolic transmural pressure did not fall, because a slight and nonsignificant rise in systolic blood pressure offset it [25]. Output fell instead: stroke volume index from 34.2 ± 1.7 to 30.5 ± 1.0 mL/m^2^, *p* < 0.01, and cardiac index from 2.20 ± 0.14 to 1.96 ± 0.14 L/min/m^2^, *p* < 0.025 [25]. The unloading arm exists in both groups; what differs is whether it is banked, and where the ventricle is preload responsive the falling stroke volume drives an arterial response that cancels it. Alviar and colleagues state the same rule from the opposite direction, that at normal or low wedge pressure positive end-expiratory pressure may lower cardiac output [17], resting it on the data quoted next. The heart failure guideline carries the same caution, that raising intrathoracic pressure decreases venous return and biventricular preload and should be used with caution where preload reserve is reduced [26], and rests it on the review already cited [8]. That harm would fall on the modest infarct with preserved ejection fraction, masked and then laid flat for angiography.

The rule has been measured in people three times, and the earliest measurement included the population this paper is about. Grace and Greenbaum stratified 21 mechanically ventilated patients with left ventricular dysfunction by wedge pressure [27], 15 of them with acute myocardial infarction and 8 of those in cardiogenic shock. Below 14 mmHg cardiac output fell in three of four data sets, between 14 and 18 mmHg it rose in four of six, and at or above 19 mmHg performance improved in twelve of thirteen by a mean of 500 mL/min [27]. The end-expiratory pressure that maximized output rose across the three strata from 0.75 to 2.0 to 3.9 cmH_2_O [27]. Bradley and colleagues split 22 patients with dilated cardiomyopathy at a wedge pressure of 12 mmHg: on 5 cmH_2_O cardiac index rose from 2.48 ± 0.26 to 2.82 ± 0.26 L/min/m^2^ above the threshold, *p* < 0.01, and fell from 3.14 ± 0.44 to 2.89 ± 0.62 L/min/m^2^ below it, *p* < 0.025 [28]. De Hoyos and colleagues added a dose. At 5 and then 10 cmH_2_O above the threshold, cardiac index rose to 2.91 and then 3.12 L/min/m^2^, and stroke volume index from 27.8 ± 3.9 to 33.9 ± 4.2 and then 36.8 ± 5.5 mL/m^2^, a rise of 9.0 mL/m^2^ [29]. Below the threshold, and in five subjects with normal ventricles, both fell stepwise [29].

Inside the high stratum the rule goes quiet, and that is its own prediction. De Hoyos found no change in cardiac index in the six patients whose wedge pressure lay between 8 and 12 mmHg, while all five below 8 mmHg fell and all five above 12 mmHg rose [29]. Bradley found the response above 12 mmHg flat rather than linear [28], so a cohort recruited entirely from above the threshold cannot show a gradient. Chadda and colleagues recruited six patients from well above that threshold, and on 10 cmH_2_O transmural occlusion pressure fell from 29 ± 4 to 15 ± 2 mmHg while cardiac index moved from 2.34 ± 0.18 to 2.19 ± 0.17 L/min/m^2^, *p* = 0.080 [19]. Their own reading is the one to quote: they entered patients at or above 18 mmHg to maximize the chance of an increase and describe a tendency to decrease [19]. At the top of the curve the subtraction is therefore neither clearly banked nor clearly paid, and the ventricle in question was a chronic one in acute decompensation.

Two boundaries close the account. Where contractility is preserved, the ventricle is preload responsive and loses stroke volume when venous return falls, and where it is reduced, it is afterload dependent and does not [3]. In pigs, raising end-expiratory pressure from 5 to 15 cmH_2_O left cardiac output unchanged in left ventricular cardiogenic shock, *p* = 0.339, while in the healthy state wall stress fell, reaching significance at 10 cmH_2_O, and cardiac output and stroke volume fell at 15 cmH_2_O [30]. Unloading and lost output arrived together, though wall stress is not the systolic transmural pressure specified in Table 1; the finding is consistent with the construct because the healthy pig fills at normal pressure, where the rule predicts payment. What none of the evidence covers is a mask in acute coronary syndrome in a patient waiting for reperfusion (Section 8). That transfer is the inference this paper asks the reader to weigh, and the first account it can be checked against is the subendocardium.

### 4.4. The Subendocardial Ledger

Unloading is credited to a specific account. Left ventricular subendocardial perfusion is a diastolic transaction: driving pressure is diastolic aortic pressure minus left ventricular end-diastolic pressure, so a fall in end-diastolic pressure widens the gradient across a bed compressed throughout systole. Hoffman and Buckberg framed that balance as the diastolic pressure-time index, supply set by the area under the diastolic gradient, demand by the tension-time integral of systole [31]. Slowing the heart lengthens diastole and adds to the same side, so the fall of 5.2 beats per minute is not trivial [25].

The ledger has two sides. If the fall in preload lowers diastolic aortic pressure, a fall in diastolic aortic pressure may offset the gain in perfusion pressure produced by a fall in LVEDP. That is the reversal of Section 4.3 seen from the coronary side, and the subendocardium of an acutely ischemic territory is the bed least able to absorb it. The same subtraction reaches a second venous bed.

### 4.5. The Cardiorenal Axis

The kidney reads pressure through its veins. Raising pleural pressure raises right atrial pressure as recorded against the atmosphere, and the renal vein, outside the thorax, takes that increase in full. In a mixed intensive care cohort of invasively ventilated patients from the MIMIC-III database, central venous pressure rose and mean perfusion pressure fell across rising categories of positive end-expiratory pressure, and higher expiratory pressure was associated with worsening renal function in one statistical model and not in another from the same analysis [32]. If the left ventricle unloads, right atrial pressure may instead drop below baseline, a sequence inferred rather than measured, and which mechanism predominates depends upon volume status. One branch remains outside the sign rule.

A red-cell effect of correcting hypoxemia after infarction rests on one single-arm study without a control group; it is unproven and carries none of the argument here [33]. The two cardiac submechanisms return to the pressure side, the first of them mitral.

### 4.6. Ischemic Mitral Regurgitation from the Circumflex

Circumflex ischemia renders the lateral and posterior wall dyskinetic. That regional displacement, and not failure of the papillary muscle itself, tethers the mitral leaflets and drives dynamic, afterload-dependent regurgitation [34,35,36].

Two experiments fix that geometry. Messas and colleagues added papillary ischemia to established inferior ischemia in sheep, and the leak decreased rather than worsened, regurgitant fraction falling from 25.2 ± 2.8% to 7 ± 1.5% [37]. Otsuji and colleagues ligated the circumflex acutely in dogs and found that despite an ejection fraction of 23 ± 8% regurgitation was trace while dilation was limited, becoming moderate only once prominent dilation followed [38]; Piérard and Lancellotti showed the other dependence during exercise echocardiography, where ventricles of similar size diverged in regurgitant volume with loading conditions alone [36]. Every link requires a dilated ventricle, which bounds the hypothesis rather than dissolving it.

The relationship between applied pressure and the leak has been measured directly in people, although never in the case described in this section. In 50 patients under general anesthesia for edge-to-edge mitral repair, raising end-expiratory pressure from 3 to 20 mmHg shortened the ventricle, narrowed the mitral annulus, and lengthened leaflet coaptation, all *p* < 0.001, most of whom had functional or mixed regurgitation [39]. In a randomized trial of 17 patients with chronic heart failure and Cheyne-Stokes respiration, three months of nocturnal continuous positive airway pressure lowered regurgitant fraction by a mean of 13.3 ± 4.6 percentage points, compared with a rise of 2.8 ± 2.6 with medical therapy alone, *p* < 0.01 [40]. That trial excluded recent infarction and unstable angina, so it says nothing about an ischemic leak [40]. Withdrawal of ventilation runs the other way: spontaneous breathing raises occlusion pressure in obstructive lung disease with coexisting cardiac disease [41], and echocardiography during weaning records the mitral regurgitation that accompanies that rise [42,43]. Nobody has recorded the ischemic leak of circumflex origin under applied pressure. The second submechanism moves the argument to the right ventricle.

### 4.7. Ventricular Interdependence in Right Ventricular Involvement

Three pathways lead to benefit, and two of them also cause harm. Every one scales with the degree of heart failure present before the mask goes on. The first reduces right ventricular preload by reducing venous return. Right ventricular wall stress falls, and with it the septum’s movement toward the left ventricle [44], so the left ventricle fills better and the measured LVEDP decreases. If LVEDP falls far enough, it may widen the coronary perfusion gradient, a step inferred rather than measured. Where the left ventricle is already underfilled and preload responsive, the same fall in venous return is inferred to cost output instead, the dissociation that Table 1, row 3, tests. The second pathway relies on lung volume. Beyond a certain volume, additional inflation increases the right ventricle’s afterload and the situation worsens [17]; short of that point, inflation decreases the afterload the right ventricle meets and its performance may improve. A third route runs through systolic coupling rather than diastolic, and it is usually left out. As the left ventricle contracts, it produces force against the right ventricular wall and may add to the force the right ventricle produces itself. Direct data in this context are absent, but canine studies attribute 20% to 40% of right ventricular systolic pressure to left ventricular contraction [45].

### 4.8. A Shared Metric

Two branches of the applied pressure share one unit, the swing in esophageal pressure, which sets left ventricular transmural pressure on one side and lung stress on the other.

The two readings start from very different baselines. In acute cardiogenic pulmonary edema, the negative esophageal pressure swing begins at 15.2 ± 1.9 cmH_2_O in nine patients with dilated cardiomyopathy in New York Heart Association class IV, and 10 cmH_2_O of applied pressure narrows it to 10.8 ± 1.9 cmH_2_O, *p* < 0.001 [22]. A companion study of six patients found the same direction without significance, continuous pressure at 5 and 10 cmH_2_O reduced the swing by 19% and 17%, against 37% for bilevel support, the only significant reduction, *p* = 0.02 [19].

The effect on effort is therefore secure in direction and not in size. In de novo hypoxemic respiratory failure, effort begins at a median tidal swing of 34 cmH_2_O, and a reduction of at least 10 cmH_2_O over two hours was the best predictor of avoiding intubation, OR 15 (95% CI 2.8 to 110) [46]. That source states the threshold two ways inside its own results, as a reduction of 10 cmH_2_O or more and as a swing below 10 cmH_2_O [46]. A decrement calibrated on a baseline twice as high does not carry across. Only the absolute level plausibly translates, because patients on effective pressure converge at approximately the same swing, 10.8 cmH_2_O in cardiogenic pulmonary edema and a median of 11 cmH_2_O at two hours in the de novo patients who avoided intubation [22,46]. A testable proposition, not a bedside rule.

## 5. What the Trial Record Can and Cannot Say

This is a narrative review, not a systematic one. I searched PubMed, Embase, the Cochrane Central Register of Controlled Trials and Web of Science from inception to 26 August 2026 and hand-searched the reference lists of the anchor synthesis and of prior meta-analyses. I kept studies that measured a hemodynamic or gas-exchange effect of applied airway pressure, or that reported a randomized comparison in acute cardiogenic pulmonary edema, with no restriction by language or year and no attempt at completeness. I registered no protocol, screened in duplicate nowhere, and ran no risk-of-bias assessment of my own, so selection was purposive rather than reproducible, and every pooled figure here is transcribed from the systematically assembled synthesis [1], or, where the text says so, from the guideline reading an overlapping evidence base [4]. Of its 24 included trials, seven state a coronary exclusion in one of four forms in their entry criteria: acute infarction requiring thrombolysis or primary angioplasty, persistent ST-segment elevation, suspected ST-elevation acute coronary syndrome, or any requirement for a lifesaving or emergency intervention [1,2]. The guideline records that patients in cardiogenic shock have been almost universally excluded and that many trials also excluded patients requiring acute revascularization [4]. An earlier meta-analysis counted 34 studies, including head-to-head comparisons with no standard-care arm [47]. One of the sources used here is my own previously published meta-regression, identified as mine at each point of use, cited for the direction of a between-trial association and never as a pooled effect estimate; the priority for the underlying hypothesis belongs to the earlier independent work of Weng and colleagues [13,14].

First, the two hard endpoints behave differently; the grading carries the argument. Moderate certainty attaches to the intubation reduction and low certainty to the mortality estimate, downgraded once for serious risk of bias and once for imprecision [1]; Section 1 gives both with their intervals. An estimate resting on trials with unclear allocation concealment and few events is not stable enough to average, and a fragile signal may reflect an artifact. The same evidence carries a recommendation: the heart failure guideline advises non-invasive ventilation at class IIa B, resting it on the review already cited and on the analysis of Weng and colleagues, while recording in its text that the largest trial was neutral [2,8,13,26]. The construct is not the only reading of that attenuation. Smaller trials at higher risk of bias tend to report larger effects. Co-interventions and background therapy changed across the two decades the pooled trials span. Intubation in the control arm of the largest trial was rare, which leaves little room for a difference, and crossover between arms blurs the contrast. The restricted synthesis rests on 7 trials, so its interval is wide. Any of these can produce the pattern the construct explains, and none of them is excluded by the data. I offer the sign rule as one candidate explanation, testable between strata, and not as the resolution of the discrepancy between symptomatic improvement and mortality.

Second, coronary safety, which a paper arguing for pressure in coronary patients cannot report selectively. The pooled estimate for myocardial infarction shows probably little or no difference, and should not be quoted without noting that infarction was defined inconsistently and median follow-up was three days [1]. The respiratory guideline reported a numerical increase, OR 1.18 (95% CI 0.95 to 1.48) at very low certainty, in the same justification that recorded a mortality reduction of RR 0.80 (95% CI 0.66 to 0.96) at moderate certainty, then withheld its recommendation from acute coronary syndrome and cardiogenic shock [4]. Reassurance and exclusion come from one page, and citing either alone misrepresents the source. Behind both sits an older literature [8]. One trial comparing bilevel support with continuous pressure stopped early after 27 patients because infarction was more frequent in the bilevel arm, although most of those patients already had chest pain on admission. A second reported infarction in 55% against 10% and intubation in 80% against 20%, under a protocol that imposed very low pressure support and less intravenous therapy [8]. Neither establishes harm. Both establish that the question was once open and that today’s reassurance is short-horizon.

Third, the boundary. What plausibly separated benefit from no benefit was not the coronary label, which the trials carried in force, but the requirement for emergency reperfusion, which was an explicit exclusion criterion by the largest trial [2,4]. No trial tested that contrast directly, so the boundary is read off exclusion criteria rather than measured. The coronary label itself was tested once, and the test was null: in an exploratory subgroup analysis, the largest trial found no interaction between treatment effect and the presence or absence of myocardial infarction at presentation, *p* = 0.93 [2]. That result runs outside the region this paper claims as its own, since the same trial had already excluded every patient who needed an emergency intervention [2]. The consequence is symmetrical: the largest trial stops being an argument for the construct and stops being an argument against it. The guideline withholds its recommendation from acute coronary syndrome and cardiogenic shock; that limits its scope and reports no finding [4]. The review lists isolated right ventricular failure among the relative contraindications and refractory hypotension among the absolute ones, which restricts the patient rather than the setting [8]. The short-horizon record carries no harm signal. Nothing here argues against a mask worn while a patient is prepared for reperfusion, so long as it delays neither the artery nor the tube.

At the other extreme sits a small trial in edema complicating infarction, itself one of the 24 pooled in the anchor synthesis, with a large mortality difference on a handful of events and unrandomized intubated patients [1,48].

## 6. The Ceiling and the Floor

Unloading has an upper boundary, and it is hemodynamic rather than pulmonary. Up to 80% of patients in cardiogenic shock develop respiratory failure, yet the limiting substrate sits in the circulation [17], and airway pressure cannot restore perfusion that has failed for want of forward flow.

Registries locate that ceiling, and they show best what clinicians declined to do. In AltShock-2, patients on oxygen or on a mask were concentrated in the less severe shock stages and those on invasive ventilation in the most severe [49], and a second cohort confines any advantage to the least severe stratum [50]. None of this compares treatments: the intubated patient was sicker, and in at least two registries the exposure was assigned by the highest intensity of support used during the admission, so patients who deteriorated on a mask counted as invasive [49,51]. One observation survives because it compares like with like: among ventilated patients in FRENSHOCK, 30-day mortality did not differ between the 237 intubated directly and the 44 intubated after a trial of non-invasive support, 31.7% against 31.8%, *p* = 0.899 [51]. On 44 patients that excludes little, so it is limited reassurance against the charge that the mask kills by delaying the tube, and the only registry finding I carry forward.

Access to reperfusion tracks outcome here, and confounding by indication leaves causation unrecoverable. In national inpatient data, patients on no ventilatory support were revascularized far more often than those on non-invasive ventilation, whose adjusted mortality was higher [52]. A separate ST-elevation population repeats it, and the authors read access rather than harm: ventilated patients received fewer revascularization procedures and may have died before reaching them [53]. A patient stable enough to lie flat for intervention is not the patient masked in extremis.

Beneath all of it lies the microvascular bed, and it sets the floor of what any airway pressure can reach. In a pooled analysis of 1688 patients imaged by cardiac magnetic resonance within seven days of primary intervention, microvascular obstruction was present in 960, or 56.9%, and its extent tracked mortality in graded fashion, adjusted hazard ratio 1.14 (95% CI 1.09 to 1.19) per 1.0% absolute increase [54]. Once mean coronary perfusion pressure falls below 45 mmHg, maximally dilated arterioles no longer hold capillary hydrostatic pressure and pericytes constrict, a derecruitment reversible early and permanent late, and established no reflow is treated in part by raising distal coronary pressure to recruit the bed [55]. Airway pressure reaches the same variable from the other end, since lowering left ventricular end-diastolic pressure widens the diastolic gradient across that bed while any fall in systemic pressure narrows it. Which of the two dominates has not been measured.

Figure 2 places that floor under the window and the ceiling above it.

## 7. From Physiology to the Bedside

The chest film discriminates poorly here. What separates a patient off support by morning from one slowly failed by a mask is rarely the radiographic pattern of congestion. It is read in three cheap signals: arterial gas pointing to the phenotype without settling it; troponin indicating myocardial injury, with a coronary cause assessed from the clinical presentation, electrocardiography, and imaging; and the hemodynamic snapshot showing how close shock sits. The gas carries the least of the three, since the two distributions overlap across most of their range and no cut point assigns an individual patient to a phenotype (Section 3) [10]. Filling pressure is not among these signals either. Non-invasively, the bedside algorithm reports left atrial rather than end-diastolic pressure [56]; across 94 studies, the mitral inflow-to-annular velocity ratio correlates with end-diastolic pressure at 0.50 (95% CI 0.38 to 0.60), and the interval reaches zero when ejection fraction is below 50% [57].

The physiology of Section 4 becomes operational there. A eucapnic or frankly hypocapnic presentation with preserved systemic pressure is where transmural unloading plausibly does its hardest work, and recruitment is not the discriminating part, since it improves gas exchange in both [23]. The unloading is banked only where the ventricle is not preload responsive.

Applied pressure does not open the artery, and I found no study showing that it limits infarct size. It can hold gas exchange and loading conditions in a range that lets reperfusion happen at all, without an endotracheal tube. Giordana and colleagues applied continuous positive airway pressure during angioplasty in 25 patients with acute myocardial infarction [58]. They tested feasibility and safety. Their series shows that the support can be delivered in the catheterization laboratory, not that it changes outcome. Such a conduit registers on a mortality scale only when what it prevents is lethal or blocks definitive treatment, and only the second has support here, indirectly (Section 6). A bridge effect is not a prognosis-modifying therapy; it predicts that any survival benefit should shrink wherever reperfusion is unavailable or already assured.

Now the other side of the ledger, where one harm hides behind a reassuring saturation. A patient whose drive stays high generates large negative pleural pressure swings of his own, and in acute respiratory distress syndrome, where the concept was developed, those swings injure the lung whatever the oximeter reads [59]. Failure of inspiratory effort to fall over the first hours tracked with intubation in de novo hypoxemic respiratory failure [46], a cohort that excluded acute cardiogenic pulmonary edema; here the driver of effort is a ventricle that positive pressure also unloads, so effort and its cause can fall together. Bedside scores that predict failure of non-invasive support, such as HACOR, were derived and validated in other forms of respiratory failure [60], so their use here is a transfer.

Two scenarios show what is easy to miss. The first is the silent circumflex, often mute on the electrocardiogram: a substantial proportion of acute occlusions show no diagnostic ST-segment change [61,62]. Flash pulmonary edema may then be the first sign, and the mask that relieves it can become the reason angiography is deferred. The dynamic regurgitation of Section 4.6 belongs here only where the ventricle has already remodeled. The urgent signal is the catheter.

The second scenario cuts both ways. In inferior ST-elevation infarction with right ventricular involvement and hypotension, applied pressure is double-edged for the reasons set out in Section 4.7, and the review restricts its use further, as Section 5 sets out [8]. The review’s general starting level of 5 cmH_2_O is not a setting for this subgroup [8], and physiological rhythm comes first because biventricular output is rate- and synchrony-dependent [44].

Two boundaries close the section. Guidelines recommend either mode in cardiogenic pulmonary edema without stratification by carbon dioxide [4], so phenotype modifies expected benefit, not the escalation threshold; the review covering coronary patients stops at what the Fifth Universal Definition now classifies as secondary myocardial infarction and at non-ST-elevation presentations [8,15]. The peri-procedural use reasoned toward here lies beyond that line: hypothesis, not recommendation.

I set out no trial design here. The comparator, the detailed escalation rules, the definition of treatment failure and the sample size belong in a protocol, and this paper stops at the predictions. For any protocol in acute coronary syndrome with pulmonary edema, the mask must not delay the invasive strategy, which the 2023 ESC guideline recommends immediately when acute heart failure is presumed secondary to ongoing myocardial ischemia, even in a non-ST-elevation presentation [63]. Nor may intubation be withheld for the sake of the study once the criteria used in acute heart failure are met, among them progressive deterioration of pH or blood gases despite non-invasive ventilation, worsening consciousness, and persistent hemodynamic instability [8].

Two of the refuting observations in Table 1 mark the points where the construct parts company with a rival reading. In row 1, if filling pressure modified nothing, the effect would be uniform and both results listed there would follow; either one is enough to refute the construct. Row 3 tests the reading based on filling pressure alone, which expects stroke volume to rise wherever end-diastolic pressure is high, dilated right ventricle or not; this row separates that reading from the construct. Row 2 asks for no more than the direction already measured in other populations. Row 4 can only constrain the construct, because the access data behind it are observational. The first three rows record hemodynamics, not deaths, since the claim is mechanistic. Each row is read against no difference with a 95% CI, because I found no clinically validated margin; an interval that still admits the predicted effect leaves the row undecided.

## 8. Limitations

Four limits bear on the argument, beyond the purposive selection and the low certainty of the pooled mortality estimate.

First, the central mechanism has never been measured in the population this paper is about. No study has recorded left ventricular transmural pressure under positive airway pressure in an acute coronary syndrome, and the transfer from chronic heart failure is an analogy, modest in size, drawn from patients who were normoxic, eucapnic, not in pulmonary edema, and whose filling pressures were not measured. Filling pressure comes instead from three stratified studies, one in a cohort where 15 of 21 patients had acute myocardial infarction [27] and two in dilated cardiomyopathy [28,29]; none applied a face mask in acute pulmonary edema, and none enrolled a patient waiting for reperfusion. Only the first includes the coronary population, and it is the weakest. Grace and Greenbaum evaluated the effect retrospectively and drew 23 data sets from 21 patients, so that one patient with infarction and shock contributed three sets falling into three different strata; they also used mixed venous oxygen tension rather than cardiac output in 2 of the 21 [27]. The other two are one research program rather than two replications, since both came from the same Toronto laboratory with shared authors, the same 12 mmHg threshold, and the same catheterization protocol [28,29].

Inside a single high-pressure stratum, filling pressure did not predict the individual response [22]; a cohort restricted to occlusion pressures of 18 mmHg or more showed no rise in cardiac output [19]; and in the chronic heart failure cohort roughly half the patients increased their cardiac index on pressure and half decreased it [25]. That split cannot be read against the refuting observation of Table 1, row 1, because Naughton and colleagues did not measure filling pressures and their patients cannot be assigned to a stratum [25]. The construct is offered at the level of strata, not patients; the inference that a reader should weigh most heavily is the transfer to the coronary patient on a mask.

Second, the phenotype signal is ecological in both readings and drawn from largely the same trials, so their agreement is not independent replication [1,13,14]. The one within-trial test of the coronary label was null; Section 5 says what that does and does not settle [2]. My own meta-regression carries a further defect, disclosed in Section 3, so I cite it for direction, not for magnitude [14].

Third, two mechanisms given substantial space have no measurement under applied pressure in the setting they describe. The mitral route is inferred from afterload reduction in a different population and from the reverse observation at weaning; the right ventricular route is double-edged, its net effect unproven. I keep both because they yield falsifiable predictions and name missing experiments.

Fourth, coronary safety rests on short-horizon data with inconsistent infarction definitions, and an older literature once raised the opposite concern [1,4,8]. Any peri-procedural use of the kind reasoned toward here lies outside current guidelines [4,8] and is a research question.

## 9. Concluding Remarks

Whether the mask saves lives is a poorly posed question, and physiology explains why. One applied pressure is not one therapy. It inflates flooded air spaces without removing lung water, can unload the left ventricle by raising surrounding pressure and thereby reducing left ventricular transmural pressure, shifts venous congestion at the kidney in a direction inferred to depend on volume state, and reaches two cardiac submechanisms where the same stimulus is either inferred or double-edged.

One rule organizes the largest of those effects. The venous return that pressure subtracts is banked where the ventricle is not preload responsive and paid where it is. Filling pressure only stands in for that state, as a candidate effect modifier. If the rule holds, benefit should concentrate in the acutely ischemic, stiff ventricle filling at high pressure whose lungs flooded in minutes, and the same pressure should cost output in the modest infarct filling normally. The rule does not govern the pulmonary arm, which follows lung volume and does not reverse. The modifier parts from the state wherever a high recorded pressure meets an underfilled, preload-responsive ventricle, in the states the introduction lists. That is why those scenarios are the sharpest test rather than exceptions.

That division offers one reading of the puzzle the introduction left open. Under the sign rule, the pulmonary and effort arms do not reverse and act alike in both phenotypes, which would leave intubation as the more secure of the two endpoints; the cardiac arm changes sign between strata, and averaging across an unstratified population can cancel part of whatever mortality effect it produces. That is one reading of the gap between the two endpoints, and Section 5 lists the others, from small-trial bias to crossover between arms, none of which the data exclude. The inference is mine and it is untested. I defend a mechanism and a hypothesis, not a proven survival effect; Table 1 states what would refute it. If the hypothesis holds, its bedside consequence would be narrow: a blood gas would not identify the phenotype, recording the gas and the troponin would let future work test the difference between groups, and an averaged estimate would say little about the patient in front of the clinician.

## Figures and Tables

**Figure 1:**
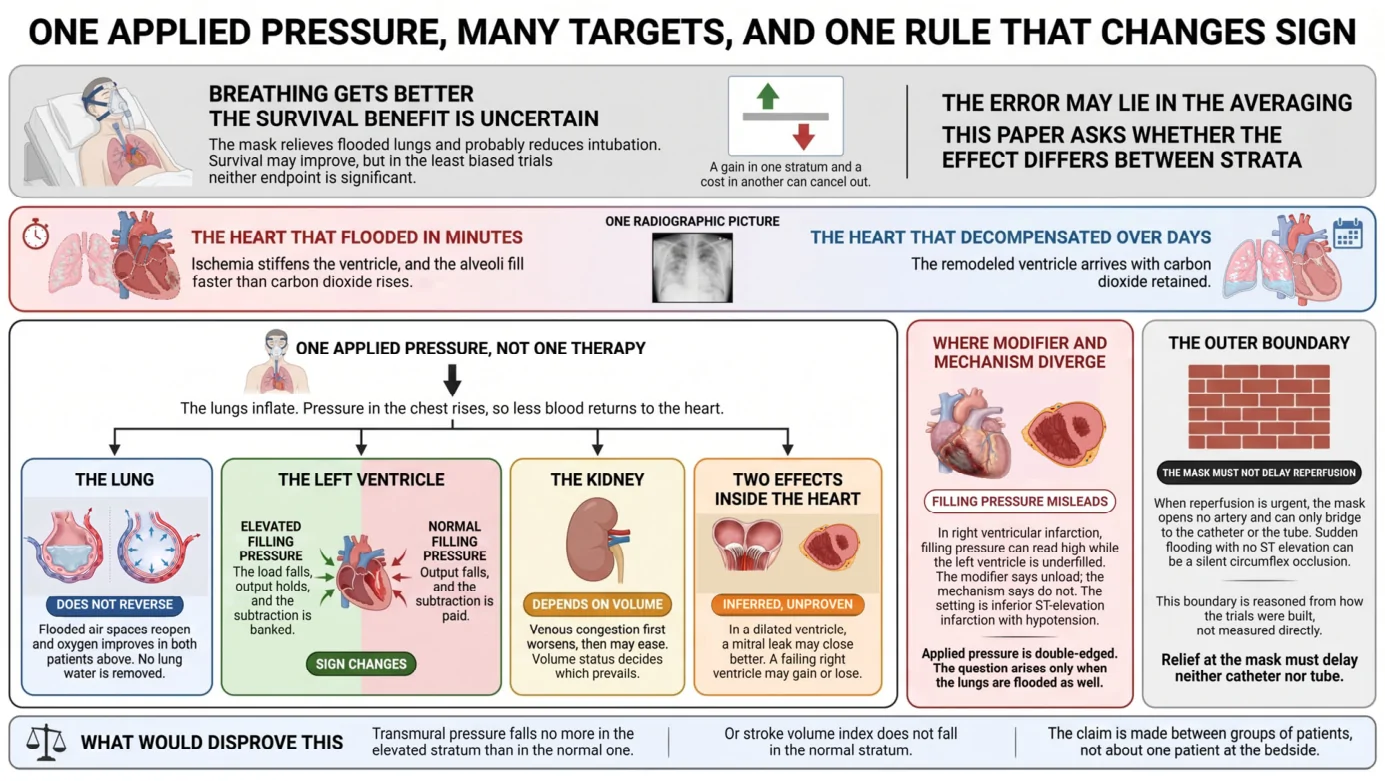
One applied pressure, many targets, and one rule that changes sign. The branches are plausible scenarios, not settled facts, and the drawing carries no line coding for the evidence tiers of Section 4.1. Black arrows trace the applied pressure to its four targets. Green and red arrows mark a gain and a cost: in the inset, between strata; in the left ventricular panel, the same pressure acting on a ventricle with elevated and with normal filling pressure. In the lung panel, blue arrows show the flooded air space reopening, and the small arrows at the vessel ends show the direction of blood flow.

**Figure 2:**
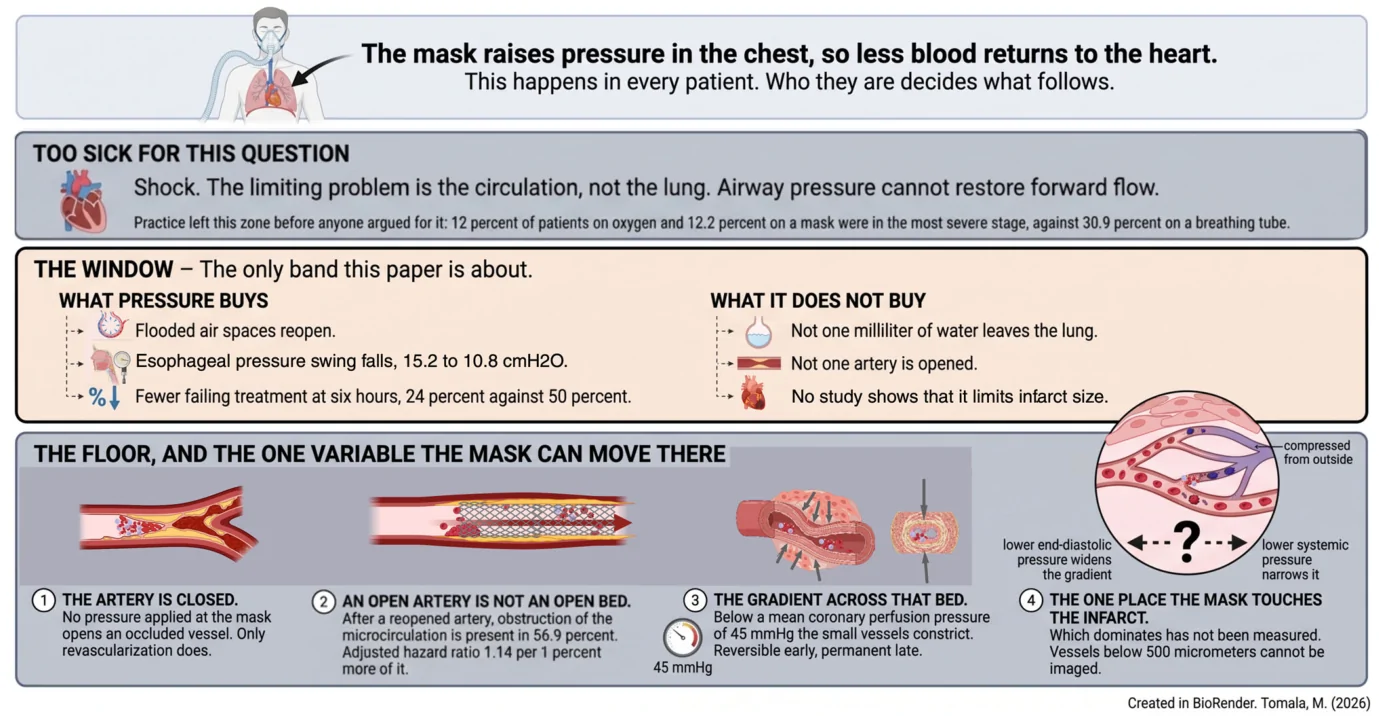
Who this question is about, what applied pressure buys, and the one variable the mask can move at the floor. Above the window, the limiting problem is the circulation rather than the lung, and practice had already left that zone before anyone argued for it, as far as registries with misclassified exposure can show (Section 6). Inside the window, applied pressure reopens flooded air spaces and narrows the inspiratory esophageal pressure swing. It removes no lung water and opens no artery. Below the window lies the microvascular bed. No pressure applied at the mask opens an occluded vessel. Coronary perfusion pressure decides whether its small vessels stay open, and applied pressure reaches that gradient from the other end: lowering left ventricular end-diastolic pressure widens it while any fall in systemic pressure narrows it, and which of the two dominates has not been measured. The figure states two values that the text does not: the shock-stage proportions from that registry, 12% of patients on oxygen and 12.2% on a mask in stage D against 30.9% on invasive ventilation, where exposure was assigned by the highest intensity of support used during the admission [49]; and the imaging limit below which vessels cannot be seen, 500 micrometers [55]. Created in BioRender. Tomala, M. (2026).

**Table 1:** Predictions of the filling-pressure construct, the quantity each requires, and the observation that would refute it. Row 1 compares filling-pressure strata because the construct claims a gradient, not a threshold; the strata are an analytic device, not a cut-off. Rows 2 and 3 each apply to the one condition they name, and row 4 to access to urgent reperfusion.

Prediction	Quantity to Measure	Refuting Observation	Evidence Level Now
Applied pressure subtracts venous return, spent as unloading where filling pressure is elevated and lost as output where it is normal.	Systolic left ventricular transmural pressure from simultaneous intracavitary (or justified arterial) and esophageal pressure; stroke volume index; left ventricular end-diastolic pressure, or a named surrogate, to assign the stratum	A fall in mean systolic transmural pressure no larger in the elevated stratum than in the normal one (elevated minus normal, 95% CI excluding values above zero); or a change in stroke volume index in the normal stratum with a 95% CI excluding a fall	Measured in another population: three stratified cohorts, one with 15 of 21 patients in acute myocardial infarction; transmural pressure and stratification never measured together.
Dynamic circumflex mitral regurgitation falls under applied pressure in ventricles already dilated.	Regurgitant fraction and effective orifice area before and during applied pressure, in dilated ventricles with a circumflex culprit.	An increase in regurgitant fraction with a confidence interval entirely above zero. An interval including zero is inconclusive, and a reduction smaller than in chronic heart failure does not refute it.	Measured in another population: under general anesthesia and in chronic heart failure.
In ischemic right ventricular involvement, left ventricular end-diastolic pressure and the mechanism dissociate, so the response follows the underfilled chamber rather than the measured filling pressure.	Septal curvature, right and left filling pressures and stroke volume index across rising applied pressure, in inferior infarction with right ventricular involvement.	A mean stroke volume index that rises with applied pressure in the stratum with elevated left ventricular end-diastolic pressure and a dilated right ventricle, with a 95% CI excluding zero.	Inferred, direction only; net effect unproven.
Survival benefit shrinks where reperfusion is unavailable or already assured.	Mortality effect stratified by access to emergency reperfusion.	A larger benefit where reperfusion is unavailable or already assured (ratio of stratum-specific risk ratios, 95% CI excluding 1.0 in that direction).	Inferred from observational access data, confounded by indication; the row constrains the construct rather than refuting it.

## Data Availability

No new data were created or analyzed in this study. All data discussed are available in the cited publications.
